# Association between perinatal complications and venous thromboembolism in postpartum women

**DOI:** 10.7189/jogh.15.04153

**Published:** 2025-05-16

**Authors:** Jing Mao, Hanxiang Sun, Qinxin Shen, Chang Zou, Yuanyuan Yang, Qiaoling Du

**Affiliations:** Department of Obstetrics, Shanghai Key Laboratory of Maternal Fetal Medicine, Shanghai Institute of Maternal-Fetal Medicine and Gynecologic Oncology, Shanghai First Maternity and Infant Hospital, School of Medicine, Tongji University, Shanghai, China

## Abstract

**Background:**

Several studies have been conducted on the risk factors for but there is a lack of research on the relationship between perinatal complications and venous thromboembolism (VTE) in large samples of Asian populations. This study aimed to systematically investigate the relationship between perinatal complications and VTE in Asian populations.

**Methods:**

This retrospective study included 40 213 women delivering singleton pregnancies. We compared the perinatal complications between the two groups, including hypertensive disorder complicating pregnancy (HDCP), preeclampsia, maternal fever before delivery, preterm birth, intrahepatic cholestasis of pregnancy, gestational diabetes mellitus, meconium-stained amniotic fluid, postpartum hemorrhage, and premature rupture of membranes. Furthermore, we conducted a logistic regression analysis to explore the relationship between VTE and the risk of adverse perinatal outcomes.

**Results:**

We observed that mothers in the VTE group were significantly more likely to have been aged 35 years or older (28.79 *vs*. 17.23%, *P* = 0.013) and to have undergone cesarean delivery (89.39 *vs*. 48.19%, *P* < 0.000) compared to the non-VTE group. Further logistic regression analysis revealed that, regardless of adjustment for confounding factors, the VTE group was significantly associated with HDCP, preeclampsia, maternal fever before delivery, preterm birth, and intrahepatic cholestasis of pregnancy, with statistically significant differences.

**Conclusions:**

Compared to the non-VTE group, the VTE group was more likely to be older (≥35 years) and exhibited a higher cesarean section rate. Additionally, the VTE group was significantly associated with increased odds of HDCP, preeclampsia, maternal fever before delivery, premature delivery and intrahepatic cholestasis of pregnancy compared to the non-VTE group, regardless of whether the confounding factors were adjusted.

Venous thromboembolism (VTE) encompasses both pulmonary embolism (PE) and deep vein thrombosis (DVT). Studies have found that the likelihood of developing DVT in women during pregnancy is five to six times higher than that in non-pregnant women [1,2]. The risk of VTE is highest in the weeks following delivery, with the first week postpartum being particularly critical [3]. Postpartum hemorrhage is the leading cause of maternal mortality in developing countries. However, in developed countries, embolic diseases are the primary cause of maternal deaths [4]. Between 2011 and 2013, pulmonary embolism was the cause of 9.2% of pregnancy-related deaths in the USA [5].

The high-risk factors for VTE included advanced age, obesity, history of VTE, immobilisation, active cancer, and smoking, among others [4,6–9]. Recent studies have highlighted additional risks in pregnancy and postpartum periods. For instance, hypertensive disorder complicating pregnancy (HDCP) and preeclampsia were independently associated with increased risks of pulmonary embolism and postpartum VTE [6,10]. Cesarean section was also linked to a higher risk of VTE compared to vaginal delivery [11,12]. Other studies have found that emergency cesarean section, compared to planned surgery, was associated with a higher risk of VTE [4,10]. In addition, in-vitro fertilisation and multiple pregnancies were also linked to a higher risk of VTE [13–15]. Infection was another recognised high-risk factor for VTE during pregnancy and the postpartum period. A cohort study in the UK showed that prenatal urinary tract infections were linked to a higher risk of VTE [16]. Other studies have identified interesting factors as well, such as the finding by Virkus et al. that pregnant women carrying male foetuses had a slightly higher risk of venous thrombosis compared to those carrying female foetuses. However, the gender of the fetus did not increase the risk of VTE after delivery [17].

The increased risk of thromboembolism during pregnancy is related to physiological and anatomical changes, including increased venous stasis, hypercoagulability, and reduced venous outflow, as well as decreased activity [18,19]. These changes, along with immune alterations like cytokine surges and endothelial dysfunction, contribute to the prothrombotic state during pregnancy and postpartum [20–24]. Currently, CT Pulmonary Angiography is an important method for diagnosing pulmonary embolism [25]. Most VTE cases can be treated with low-dose low-molecular-weight heparin, but thrombolysis and reperfusion therapies may be needed for limb- or life-threatening cases [26,27].

Currently, there are numerous studies in developed countries on the high-risk factors that elevate the likelihood of VTE during pregnancy or after childbirth, but there is a lack of research on the relationship between perinatal complications and VTE in large samples of Asian populations. Considering that racial factors may be related to VTE [28], the aim of our study is to systematically investigate the relationship between perinatal complications and VTE in Asian populations, providing theoretical and data support for future improvements in clinical management to prevent VTE in women with high-risk factors for VTE.

## METHODS

### Population

This retrospective cohort study took place at a major tertiary hospital in Shanghai, China, renowned as one of the leading prenatal care centers in the region. Approval for the study was granted by the hospital’s Institutional Review Board, which also exempted the study from the need for informed consent, given that all data utilised were fully anonymised.

**Figure 1:**
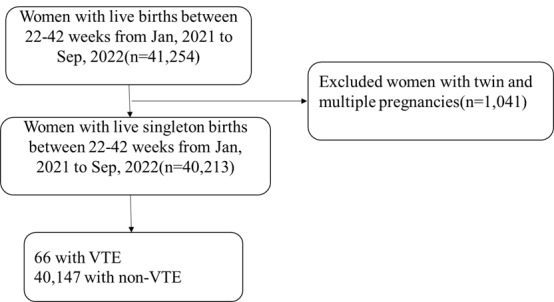
Flowchart of the process for pregnant women to join the group. VTE – venous thromboembolism.

This retrospective study reviewed 41 254 pregnant women. After excluding 1041 pregnant women with multiple pregnancies, the remaining 40 213 were pregnant women who delivered singleton pregnancies between January 2021 and September 2022, with a gestational age of 22–42 weeks. Among these 40 213 pregnant women, 66 had VTE and 40 147 did not have VTE (**Figure 1**).

### Outcome

The outcome of our study was VTE. In this study, all VTE cases were diagnosed postpartum, with no VTE cases diagnosed during pregnancy.

### Clinical variables

Demographic characteristics such as maternal age, pre-pregnancy weight and height, gravidity and parity, mode of delivery, and whether assisted reproductive technology was used during this pregnancy were also extracted from the hospital's electronic medical record system. Pre-pregnancy body mass index was calculated as weight in kilograms divided by height in meters squared.

### Exposure

The outcomes are perinatal complications, including hypertensive disorders complicating pregnancy, preeclampsia, maternal fever before delivery, premature delivery, intrahepatic cholestasis of pregnancy, gestational diabetes mellitus (GDM), meconium-stained amniotic fluid, postpartum hemorrhage, and premature rupture of membranes. Hypertensive disorder complicating pregnancy refers to a group of conditions that include gestational hypertension, preeclampsia, eclampsia, chronic hypertension with superimposed preeclampsia, and pregnancy with chronic hypertension. Preeclampsia is defined as the presence of systolic blood pressure (SBP)≥140 millimetres of mercury (mmHg) or diastolic blood pressure (DBP)≥90 mm Hg after 20 weeks of gestation, accompanied by 24-hour urine protein ≥0.3 gramme (g) or random urine protein 1+ or higher, or in the absence of proteinuria, the presence of any of the following:

1) liver dysfunction: requiring serum transaminase levels more than twice the normal value

2) renal impairment: serum creatinine levels ≥1.1 milligramme per deciliter (mg/dL) or more than twice the normal value

3) other conditions: such as thrombocytopenia, pulmonary edema, new-onset central nervous system abnormalities, or visual disturbances.

Maternal fever before delivery refers to the presence of fever before or during childbirth. Premature delivery is defined as delivery occurring at 28 weeks of gestation but before 37 weeks. Intrahepatic cholestasis of pregnancy is defined as a fasting serum total bile acid level of 10 micro millimoles per litre (μmmol/L) or higher. Gestational diabetes mellitus is defined by conducting a 75-gramme oral glucose tolerance test during pregnancy, where a fasting blood glucose level of 5.1 mmol/L or higher, a 1-hour post-glucose load blood glucose level of 10 mmol/L or higher, or a 2-hour post-glucose load blood glucose level of 8.5 mmol/L or higher, with any one of these blood glucose values meeting the criteria sufficient for diagnosing GDM. Meconium-stained amniotic fluid can be classified into three degrees based on the level of contamination. Postpartum hemorrhage is defined as bleeding of 500 ml or more in vaginal deliveries or 1000 ml or more in cesarean sections within 24 hours after the delivery of the fetus. Premature rupture of membranes refers to the spontaneous rupture of the fetal membranes before the onset of labor.

### Statistical analysis

The basic characteristics of the study population were analysed using descriptive statistics. Cross-tabulation tests were conducted to examine whether there was an association between VTE and perinatal complications (including hypertensive disorders complicating pregnancy, preeclampsia, et al) compared to parturients without VTE. A *P* < 0.05 was considered statistically significant. Logistic regression analysis was used to assess whether VTE was linked to a higher association with HDCP, preeclampsia, maternal fever before delivery, premature delivery, and intrahepatic cholestasis of pregnancy. Stratified analyses based on maternal age, mode of delivery, and pre-pregnancy BMI were performed. Confounding factors in this study included maternal age, pre-pregnancy body mass index (BMI) (<18.5 kilogrammes per square metre (kg/m^2^), 18.5 kg/m^2^–<24 kg/m^2^, 24 kg/m^2^–<28 kg/m^2^, ≥28 kg/m^2^), parity (nulliparous, multiparous), whether pregnancy was obtained through assisted reproductive technology, and mode of delivery. All analyses were performed using the SPSS 26.0 software package (SPSS Inc., Chicago, IL, USA).

## RESULTS

### Demographic characteristics of the participants

We divided the population into a VTE group and a non-VTE group based on the presence or absence of VTE. There were 66 patients in the VTE group, and the incidence rate of VTE was 0.16% in our study. The non-VTE group consisted of 40 147 individuals, accounting for 99.84%. The VTE group exhibited a notably increased likelihood of maternal age being 35 years or older (28.79 *vs*. 17.23%, *P* = 0.013) in comparison to the non-VTE group. However, no statistically significant differences in pre-pregnancy BMI, parity, or whether pregnancy was achieved through assisted reproductive technology were observed between the two groups. For parturients in the VTE group, the probability of caesarean section was significantly higher than that in the non-VTE group (89.39 *vs*. 48.19%, *P* < 0.001) (**Table 1**).

**Table 1:** Basic characteristics of the study population with VTE or no VTE

Variables	VTE, No. (%), n = 66	non-VTE, No. (%) n = 40 147	*P-*value
Maternal age, in years			
*≤35*	47 (71.21)	33 228 (82.77)	0.013
*≥35*	19 (28.79)	6919 (17.23)	
Pre-pregnancy BMI, n (%)			
*<18.5 kg/m^2^*	5 (7.58)	4928 (12.27)	0.651
*18.5 kg/m^2^-<24 kg/m^2^*	50 (75.76)	28 335 (70.58)	
*24 kg/m^2^-<28 kg/m^2^*	9 (13.64)	5559 (13.85)	
*≥28 kg/m^2^*	2 (3.03)	1325 (3.30)	
Parity, n (%)			
*Nulliparous*	52 (78.79)	31 046 (77.33)	0.778
*Multiparous*	14 (21.21)	9101 (22.67)	
Mode of delivery, n (%)			
*Vaginal delivery*	7 (10.61)	20 802 (51.81)	<0.001
*Caesarean section*	59 (89.39)	19 345 (48.19)	
Assisted reproductive technology, n (%)			
*Yes*	7 (10.61)	3081 (7.67)	0.371
*No*	59 (89.39)	37 066 (92.33)	

BMI – body mass index, VTE – venous thromboembolism

The presence of potential risk factors in the VTE and non-VTE groups We further compared the perinatal complications between the two groups and found that, the VTE group exhibited a notably increased likelihood of HDCP (16.67 *vs*. 6.15%, *P* < 0.001), preeclampsia (10.61 *vs*. 3.03%, *P* < 0.001), maternal fever before delivery (18.18 *vs*. 8.67%, *P* = 0.006), premature delivery (15.15 *vs*. 5.31%, *P* < 0.001), and intrahepatic cholestasis of pregnancy (4.55 *vs*. 1.10%, *P* = 0.038), with all differences being statistically significant. However, there were no statistically significant differences between the two groups in terms of GDM, meconium-stained amniotic fluid, postpartum haemorrhage, and premature rupture of membranes (**Table 2**). To further explore the relationship between VTE and the risk of adverse perinatal outcomes, we conducted a Logistic regression analysis. We found that without adjusting for confounding factors, the VTE group was significantly associated with increased odds of hypertensive disorders complicating pregnancy (odds ratio (OR) = 3.049; 95% confidence interval (CI) = 1.594–5.833) and an even increased odds of preeclampsia (OR = 3.798; 95% CI = 1.732–8.333). The VTE group also exhibited a notably association with maternal fever before delivery (ORs = 2.341; 95% CI = 1.251–4.381), premature delivery (OR = 3.184; 95% CI = 1.622–6.249), and intrahepatic cholestasis of pregnancy (OR = 4.268; 95% CI = 1.335–13.641), respectively (**Table 2**).

**Table 2:** The presence of potential risk factors in the VTE and non-VTE groups

Variables*	VTE, No. (%), n = 66	non-VTE, No. (%), n = 40 147	*P-*value	Crude OR (95% CI)†
Gestational diabetes mellitus, n (%)				
*Yes*	8 (12.12)	5536 (13.79)	0.694	0.862 (0.412–1.807)
*No*	58 (87.88)	34 611 (86.21)		
Hypertensive disorder complicating pregnancy, n (%)				
*Yes*	11 (16.67)	2471 (6.15)	<0.001	3.049 (1.594–5.833)
*No*	55 (83.33)	37 676 (93.85)		
Preeclampsia, n (%)				
*Yes*	7 (10.61)	1216 (3.03)	<0.001	3.798 (1.732–8.333)
*No*	59 (89.39)	38 931 (96.97)		
Meconium-stained amniotic fluid, n (%)				
*Yes*	8 (12.12)	7379 (18.38)	0.190	0.613 (0.292–1.283)
*No*	58 (87.88)	32 768 (81.62)		
Maternal fever before delivery, n (%)				
*Yes*	12 (18.18)	3480 (8.67)	0.006	2.341 (1.251–4.381)
*No*	54 (81.82)	36 667 (91.33)		
Premature delivery, n (%)				
*Yes*	10 (15.15)	2132 (5.31)	<0.001	3.184 (1.622–6.249)
*No*	56 (84.85)	38 015 (94.69)		
Postpartum haemorrhage, n (%)				
*Yes*	0 (0)	364 (0.91)	1.000	0.000
*No*	66 (100.00)	39 783 (99.09)		
Premature rupture of membranes, n (%)				
*Yes*	12 (18.18)	6771 (16.87)	0.775	1.095 (0.586–2.049)
*No*	54 (81.82)	33 376 (83.13)		
Intrahepatic cholestasis of pregnancy, n (%)				
*Yes*	3 (4.55)	443 (1.10)	0.038	4.268 (1.335–13.641)
*No*	63 (95.45)	39 704 (98.90)		

CI – confidence interval, OR – odds ratio, VTE – venous thromboembolism

*N (%).

†Crude odds ratios and 95% confidence intervals.

### Adjusted OR (95% CI) for the associations between VTE and perinatal complications

Subsequently, when accounting for confounding factors such as maternal age, pre-pregnancy BMI, and parity, whether pregnancy was obtained through assisted reproductive technology, and mode of delivery, we found that the risks of several adverse outcomes persisted and were statistically significant (**Table 3**). Specifically, the VTE group exhibited a notably association with HDCP (OR = 2.308; 95% CI = 1.179–4.517) and preeclampsia (OR = 2.574; 95% CI = 1.156–5.730), respectively. The VTE group also had higher ORs and 95% CIs for maternal fever before delivery (OR = 2.823; 95% CI = 1.480–5.385), premature delivery (OR = 2.520; 95% CI = 1.279–4.964), and intrahepatic cholestasis of pregnancy (OR = 3.259; 95% CI = 1.014–10.472) (**Table 3**) We also stratified the analysis based on maternal age, mode of delivery, and pre-pregnancy BMI, and found that the association between pregnancy complications and VTE did not change in strength with stratification. (Table S1–3 in the **Online Supplementary Discussion**).

**Table 3:** Adjusted OR (95% CI) for the associations between VTE and perinatal complications

Variables	VTE	non-VTE
Hypertensive disorder complicating pregnancy		
Adjusted*	2.308 (1.179–4.517)	Reference
Preeclampsia		
Adjusted*	2.574 (1.156–5.730)	Reference
Maternal fever before delivery		
Adjusted*	2.823 (1.480–5.385)	Reference
Premature delivery		
Adjusted*	2.520 (1.279–4.964)	Reference
Intrahepatic cholestasis of pregnancy		
Adjusted*	3.259 (1.014–10.472)	Reference

VTE – venous thromboembolism

*Adjusted according to the maternal age, pre-pregnancy body mass index, parity (nulliparous, multiparous), whether pregnancy was obtained through assisted reproductive technology, and mode of delivery (vaginal delivery, caesarean section).

## DISCUSSION

This is a large retrospective study conducted in Shanghai, China. After analysing 40 213 pregnant women with singleton pregnancies, we found that the VTE group was significantly associated with increased odds of HDCP, preeclampsia, maternal fever before delivery, preterm birth, and intrahepatic cholestasis of pregnancy, both before and after adjusting for confounding factors. Although previous studies have explored the high-risk factors for VTE during pregnancy and postpartum, our study provides data support for the high-risk factors of postpartum VTE in Asian populations.

Previous studies have shown that HDCP were high-risk factors for VTE [29,30]. A Dutch nationwide cohort study with an average follow-up time of 13.7 years found that women with HDCP and preeclampsia had a higher risk of VTE during pregnancy, postpartum, and even 13 years later [31]. An observational cohort study in Denmark from 1997 to 2016 found that preeclampsia was significantly associated with a higher risk of VTE during pregnancy, the puerperium, and postpartum, both before and after adjusting for confounding factors [32]. A study in Sweden from 1990 to 1993 found that women with preeclampsia had a 3-fold increased risk of VTE postpartum. Our study found that, without adjusting for confounding factors, the risk of HDCP was 3.049 times higher in the VTE group, and the risk of preeclampsia was even higher, at 3.798 times. After adjusting for confounding factors, women with preeclampsia had a 2.574-fold increased risk postpartum, and the risk of HDCP was 2.308 times higher. These findings were roughly consistent with the results of the Swedish study [11]. Both American and international guidelines uniformly recommend that women should be assessed for VTE risk factors in early pregnancy or before pregnancy [33]. Currently, China has implemented this recommendation from the guidelines, and we focus on assessing the risk of VTE in pregnant women with complications such as preeclampsia. Some studies have suggested that the strong association between preeclampsia and thromboembolism is multifactorial, involving endothelial cells, platelets, adhesion ligands, coagulation, and fibrinolysis [34,35]. During the systemic inflammatory process in patients with preeclampsia, haemostatic cells and factors underwent oxidative modification [34]. However, further research is needed to explore the specific molecular mechanisms underlying the association between preeclampsia and VTE.

Not all studies have considered preterm birth as one of the targets for exploring high-risk factors for VTE, so there is limited research both domestically and internationally on whether preterm birth is a high-risk factor for VTE, and there is no definitive conclusion. A study by Sultan et al. on the UK population found that women with preterm birth had a significantly higher risk of developing VTE after delivery [16]. However, a study in Wuhan, China, after adjusting for confounding factors, did not consider preterm birth to be one of the high-risk factors for VTE [30]. The findings of our study indicated that preterm birth was also among the high-risk factors contributing to VTE, with a 2.520-fold increased risk compared to the non-VTE group. A possible reason for the increased risk of VTE in women with preterm birth may be the use of progesterone-based tocolytic drugs to maintain pregnancy.

Similarly, not all studies have considered GDM as a focus for investigating risk factors that increase the likelihood of VTE. In existing research, there is no clear conclusion on whether GDM is a high-risk factor for VTE. A study in Finland found that GDM was associated with an increased risk of postpartum VTE [3]. Other studies have also found a link between GDM and thrombosis [4,36,37]. However, a study by Won et al. did not consider GDM as a high-risk factor for VTE [38]. Our study also did not find a statistically significant difference in the complication of GDM between the VTE and non-VTE groups. Therefore, more rigorous prospective studies are needed in the future to further explore the relationship between GDM and VTE. Currently, no studies have explored whether maternal fever before delivery and intrahepatic cholestasis of pregnancy are high-risk factors for VTE. Our study found that the VTE group had significantly increased odds of maternal fever before delivery (OR = 2.341; 95% CI = 1.251–4.381) and intrahepatic cholestasis of pregnancy (OR = 4.268; 95% CI = 1.335–13.641), respectively. After adjusting for confounding factors, the risks increased by 2.823 times and 3.259 times, respectively. The high risk of VTE associated with maternal fever before delivery may be related to puerperal infections, which can increase the risk of thrombosis. However, further basic research is needed in the future to explore the association between intrahepatic cholestasis of pregnancy and VTE. Although our study provides data support for the high-risk factors of postpartum VTE in Asian populations, there are also some limitations. First, the retrospective nature of the study limits its ability to establish a causal relationship between perinatal complications and VTE. Second, although the study performed logistic regression analysis to adjust for confounding factors, there may still be unmeasurable confounders that could affect the relationship between perinatal complications and VTE. For example, lifestyle factors such as physical activity, diet, and smoking status. These unmeasurable confounders could lead to spurious associations or underestimation of the true effect size. Third, although the study included a relatively large sample size of 40 213 women, it still focused on a single Asian population. Due to potential differences in genetics, cultural practices, health care systems, and environmental factors, these results may not be generalisable to other Asian populations or non-Asian populations. Finally, due to the nature of retrospective studies, data bias and missing data were inevitable. In the future, we can conduct large-sample prospective studies to avoid these limitations.

In conclusion, our analysis identified significant associations between HDCP, preeclampsia, maternal fever before delivery, preterm birth, and intrahepatic cholestasis of pregnancy with postpartum VTE. These findings have important policy implications. Healthcare providers should be vigilant about the risk of VTE in pregnant women, especially those with advanced age, HDCP, preeclampsia, maternal fever before delivery, preterm birth, and intrahepatic cholestasis of pregnancy. Implementing targeted screening and preventive measures for these high-risk groups could help reduce the incidence of postpartum VTE. Future research should focus on further exploring the underlying mechanisms linking these risk factors to VTE. Additionally, prospective studies with larger sample sizes and more detailed clinical data are needed to confirm these associations and to develop more effective preventive strategies. Collaboration across different regions and populations will also be crucial to better understand the global burden of VTE in pregnancy and to identify universal risk factors.

## CONCLUSIONS

Our study found that the VTE group was likely to be advanced age (≥35 years) in comparison to the non-VTE group. At the same time, regardless of whether confounding factors were adjusted, the VTE group had significantly increased odds of HDCP, preeclampsia, maternal fever before delivery, preterm birth, and intrahepatic cholestasis of pregnancy. Our study provides data support regarding the risk factors that elevate the chances of postpartum VTE among Asian individuals.

## Additional material


Online Supplementary Document
