# Supplementary material for: Association between perinatal complications and venous thromboembolism in postpartum women
Source: J Glob Health. 2025 May 16;15:04153. doi: 10.7189/jogh.15.04153 (PMC12082260; doi:10.7189/jogh.15.04153)
Supplement: Online Supplementary Document [file jogh-15-04153-s001.pdf]

Supplement to: Mao J, Sun H, Shen Q, Zou C, Yang Y, Du Q. Association between perinatal complications and venous thromboembolism in postpartum women. J Glob Health. 2025;15:04153.

Supplementary table 1 Crude and adjusted OR (95% CI) for the associations between VTE and perinatal complications according to maternal age

	VTE	
	<35, y	⩾35, y
Hypertensive disorder complicating pregnancy		
Crude	3.375(1.575-7.231)	2.101(0.610-7.230)
Adjusted†	2.529(1.150-5.563)	1.967(0.551-7.016)
Preeclampsia		
Crude	4.080(1.611-10.336)	2.853(0.656-12.411)
Adjusted†	2.631(1.022-6.772)	2.371(0.531-10.597)
Maternal fever before delivery		
Crude	2.879(1.464-5.662)	1.261(0.168-9.477)
Adjusted†	2.824(1.425-5.596)	1.885(0.239-14.857)
Premature delivery		
Crude	1.849(0.663-5.156)	5.452(2.064-14.401)
Adjusted†	1.548(0.554-4.326)	4.965(1.870-13.178)
Intrahepatic cholestasis of pregnancy		
Crude	3.883(0.939-16.065)	5.682(0.748-43.174)
Adjusted†	2.730(0.656-11.364)	4.435(0.579-33.970)

†Adjusted according to pre-pregnancy body mass index, parity (nulliparous, multiparous), whether pregnancy was obtained through assisted reproductive technology, and mode of delivery (vaginal delivery, caesarean section).

Abbreviation: VTE, venous thromboembolism.

Supplementary table 2 Crude and adjusted OR (95% CI) for the associations between VTE and perinatal complications according to mode of delivery

	VTE	
	Vaginal delivery	Caesarean section
Hypertensive disorder complicating pregnancy		
Crude	4.167(0.501-34.653)	2.159(1.091-4.269)
Adjusted†	4.183(0.485-36.053)	2.205(1.089-4.461)
Preeclampsia		
Crude	12.215(1.466-101.798)	2.227(0.955-5.193)
Adjusted†	12.037(1.406-103.039)	2.254(0.954-5.326)
Maternal fever before delivery		
Crude	1.568(0.189-13.028)	2.764(1.433-5.334)
Adjusted†	1.188(0.143-9.889)	3.151(1.597-6.217)
Premature delivery		
Crude	0.000	2.892(1.462-5.723)
Adjusted†	0.000	2.726(1.373-5.409)
Intrahepatic cholestasis of pregnancy		
Crude	0.000	3.322(1.034-10.671)
Adjusted†	0.000	3.470(1.076-11.190)

†Adjusted according to the maternal age, pre-pregnancy body mass index, parity (nulliparous, multiparous), and whether pregnancy was obtained through assisted reproductive technology.

Abbreviation: VTE, venous thromboembolism.

Supplementary table 3 Crude and adjusted OR (95% CI) for the associations between VTE and perinatal complications according to pre-pregnancy BMI

	VTE			
	<18.5Kg/m^2	18.5 Kg/m^2-<24 Kg/m^2	24 Kg/m^2-<28 Kg/m^2	≥28Kg/m^2
Hypertensive disorder complicating pregnancy				
Crude	0.000	4.337(2.104-8.942)	0.987(0.123-7.903)	3.233(0.202-51.841)
Adjusted†	0.000	3.252(1.563-6.770)	0.731(0.090-5.907)	2.315(0.143-37.428)
Preeclampsia				
Crude	0.000	6.455(2.894-14.400)	0.000	0.000
Adjusted†	0.000	4.357(1.932-9.825)	0.000	0.000
Maternal fever before delivery				
Crude	8.165(1.360-49.016)	1.950(0.914-4.157)	2.923(0.606-14.108)	0.000
Adjusted†	9.660(1.548-60.296)	2.358(1.081-5.146)	3.825(0.739-19.805)	0.000
Premature delivery				
Crude	0.000	3.081(1.384-6.861)	4.007(0.829-19.357)	13.096(0.813-211.034)
Adjusted†	0.000	2.391(1.069-5.350)	3.680(0.753-17.994)	12.300(0.748-202.131)
Intrahepatic cholestasis of pregnancy				

Crude	16.177(1.787-146.454)	3.755(0.909-15.516)	0.000	0.000
Adjusted†	10.750(1.117-103.430)	2.851(0.687-11.835)	0.000	0.000

†Adjusted according to the maternal age, parity (nulliparous, multiparous), whether pregnancy was obtained through assisted reproductive technology, and mode of delivery (vaginal delivery, caesarean section).

Abbreviation: VTE, venous thromboembolism. BMI, body mass index.
